# Mendelian randomization studies of lifestyle-related risk factors for stroke: a systematic review and meta-analysis

**DOI:** 10.3389/fendo.2024.1379516

**Published:** 2024-11-04

**Authors:** Yi Tian, Xin Tang, Yi Liu, Shu Yi Liu

**Affiliations:** ^1^ School of Clinical Medicine, Chengdu University of Traditional Chinese Medicine, Chengdu, Sichuan, China; ^2^ Department of Communication Sciences and Disorders, MGH Institute of Health Professions, Boston, MA, United States; ^3^ General Practice, Chengdu Integrated Traditional Chinese Medicine (TCM) & Western Medicine Hospital, Chengdu, China

**Keywords:** stroke, ischemic stroke, small vessel stroke, mendelian randomization analysis, genetic epidemiology

## Abstract

**Objective:**

Stroke risk factors often exert long-term effects, and Mendelian randomization (MR) offers significant advantages over traditional observational studies in evaluating the causal impact of these factors on stroke. This study aims to consolidate and evaluate the relationships between potential causal factors and stroke risk, drawing upon existing MR research.

**Methods:**

A comprehensive search for MR studies related to stroke was conducted up to August 2023 using databases such as PubMed, Web of Science, Embase, and Scopus. This meta-analysis examines the relationships between potential causative factors and stroke risk. Both random-effects and fixed-effects models were utilized to compile the dominance ratios of various causative elements linked to stroke. The reliability of the included studies was assessed according to the Strengthening the Reporting of Observational Studies in Epidemiology incorporating Mendelian Randomization (STROBE-MR) guidelines.

**Results:**

The analysis identified several risk factors for stroke, including obesity, hypertension, low-density lipoprotein cholesterol (LDL-C), chronic kidney disease (CKD), and smoking. Protective factors included high-density lipoprotein cholesterol (HDL-C), estimated glomerular filtration rate (eGFR), and educational attainment. Subgroup analysis revealed that type 2 diabetes mellitus (T2DM), diastolic blood pressure (DBP) are risk factors for ischemic stroke (IS).

**Conclusion:**

This study confirms that variables such as obesity, hypertension, elevated LDL-C levels, CKD, and smoking are significantly linked to the development of stroke. Our findings provide new insights into genetic susceptibility and potential biological pathways involved in stroke development.

**Systematic review registration:**

https://www.crd.york.ac.uk/PROSPERO, identifier CRD42024503049.

## 1 Introduction

Stroke, a severe neurological condition, is often triggered by decreased blood flow or breakdown of vascular structures, leading to irreversible damage to neurons in the cerebrum (1). Stroke is the second most common cause of death worldwide and a significant contributor to severe disability, impacting more than 150,000 individuals each year. According to the Global Burden of Disease (GBD) study, the annual number of strokes and stroke-related deaths increased substantially between 1990 and 2019, with incident strokes rising by 70% and stroke-related deaths by 43%. Notably, it is anticipated that by 2030, the incidence of ischemic stroke (IS), one of the two major subtypes of stroke, will escalate to 4.90 million globally (2–5). Especially in low-income countries, the disease burden of stroke is even higher (6–11). In recent years, studies on the etiology of stroke have gradually revealed connections between numerous life-style and physiologic factors and their likelihood of leading to the disease (12–15). Lower educational attainment is linked to an increased risk of stroke. Obesity, especially increased body mass index (BMI) and waist circumference (WC), is an independent predictor of stroke occurrence (16). Furthermore, smoking may result in the emergence of additional cardiovascular risk factors such as dyslipidemia, and hypertension through metabolic and hemodynamic changes (17). Importantly, the presence of chronic kidney disease (CKD) dramatically increases death risk in individuals suffering from a stroke (18). However, establishing a direct causal connection between a particular cause and its associated disease is difficult due to inherent limitations in observation-based studies, like potential confounding factors and skewed information. These hindrances make proving causality a tricky task. Mendelian Randomization (MR) is a method in human genetics, using single nucleotide polymorphisms (SNPs) as instrumental variables (IVs) in genome-wide association study(GWAS) focus on exposure events (19). It is specifically aimed at elucidating the causal connections between exposure and outcomes (20). Recent research undertaking MR has turned its attention towards identifying how specific risk factors could potentially contribute to a stroke. Given the diverse nature of these studies, it becomes overwhelmingly essential to carry out comprehensive systematic reviews coupled with meta-analysis. Such evaluations are vital in bolstering the evidence that supports effective strategies in both the prevention and treatment of stroke.

This paper presents a systematic review and meta-analysis of MR studies related to stroke. We evaluate a spectrum of stroke-related risk factors, including obesity, lipid profiles, blood pressure (BP), renal function, and environmental factors. The primary outcome of this study is total stroke. Additionally, we focus on ischemic stroke (IS), defined as an episode of neurological dysfunction caused by focal cerebral, spinal, or vascular infarction, assessed in subgroup analysis (21, 22).

## 2 Materials and method

### 2.1 Literature search

The configuration and documentation of this systematic evaluation were conducted following the recommendations of the Preferred Reporting Items for Systematic Reviews and Meta-Analyses (PRISMA) guideline (23, 100) and pre-registered with PROSPERO(CRD42024503049).

In pursuit of a comprehensive scholarly investigation, a thorough search was carried out through four databases: PubMed, Web of Science, Embase, and Scopus. The databases were searched for relevant citations published from their inception to August 20, 2023, using the search terms “Mendelian randomization analysis” combined with “stroke.” The strategies used to search the databases are described in **Supplementary Table 1**. To ensure a comprehensive literature review, we carefully reviewed the references in the selected studies to identify relevant articles potentially missed in the preliminary search. Each of these articles was manually verified for relevance. When discrepancies arose among the reviewers, they engaged in deliberative discussions until a unanimous consensus was reached. Prior to the assessment using the predefined inclusion and exclusion criteria, articles were initially screened to ensure relevance and de-emphasize any irrelevant ones.

The following criteria guided the selection of studies for inclusion in the screening process:

1. International studies published up to August 2023 that utilized the MR method to investigate causal links between stroke or related phenotypes and various risk factors;

2. All studies using genetic variation to establish causal relationships regarding the impact of exposure factors on stroke outcomes;

3. All studies that includes MR in their analysis using GWAS or phenotype-wide association studies (PheWAS);

4. Studies reporting outcomes as 95% confidence intervals (CI), odds ratios (OR), and relative risks (RR), or providing raw data that can be converted to these metrics;

5. Original research articles.

The following criteria guide the exclusion of studies in the screening process:

1. Diagnosis of other types of cerebrovascular disease, such as cerebral atherosclerosis, cerebral arteritis, cerebral aneurysm, cerebral artery injury, intracranial vascular malformation, thrombosis, and cerebral arteriovenous fistula;

2. Relevant outcome indicators were not reported or data were incomplete (101);

3. Repeatedly published studies;

4. Editorials, letters to the editor, review articles, conference coverage, systematic reviews, case reports, and experimental animal studies.

### 2.2 Literature screening and data extraction

All identified literature was imported into EndNote 20 for systematic review. Two researchers independently conducted the screening process, initially reviewing titles and abstracts, followed by full-text assessments in accordance with predefined inclusion and exclusion criteria. Articles selected during this initial phase underwent a more detailed full-text review. In cases of disagreement about eligibility, a third researcher was consulted to make the final decision.

Data extraction involved several key parameters, including the first author’s name, publication year, study ethnicity, consortium responsible for stroke genomics research, exposure variables, outcome sample size, and major findings. Additionally, OR and 95% CI were extracted using various MR methods, including inverse variance weighted (IVW), weighted median estimator (WME), MR-Egger regression, simple mode, and weighted mode (see **Supplementary Table 2**).

### 2.3 Assessment of methodological quality

The methodological quality of the studies included in the meta-analysis was evaluated using a modified version of the Strengthening the Reporting of Observational Studies in Epidemiology for Mendelian Randomization (STROBE-MR) guidelines (24). We converted quality scores into percentages: studies scoring below 75% were classified as poor quality, those scoring between 75% and 85% were considered moderate quality, and studies scoring above 85% were regarded as high quality (25, 26). Two researchers independently conducted the quality assessment, and any discrepancies were resolved through consultation with a third researcher.

### 2.4 Statistical analysis

To qualify for the meta-analysis, each study had to meet specific criteria. It needed to be among at least two independent investigations focusing on the etiology of stroke or examining the causal relationships between stroke and various genetic or contributing factors. Statistical analysis was conducted using Stata 17.0 software. Outcome measures included OR values and 95% CI as effect size metrics, with a significance threshold of α=0.05. Inter-study heterogeneity was assessed using the *χ* ^2^ test, with the *p*-value and *I*² as indicators. For *I*²< 50% and *p* > 0.1, a fixed-effects model was applied. For *I*² ≥ 50%, indicating substantial heterogeneity, sensitivity and subgroup analyses were conducted to identify sources of heterogeneity. If sources of heterogeneity could not be resolved, a random-effects model was employed (27, 28). Sensitivity analysis was performed to assess the stability of the results by comparing the combined effect estimates from both random-effects and fixed-effects models. Significant differences indicated high sensitivity and potential instability in the outcomes of the meta-analysis. Egger’s test was utilized for funnel plot analysis to evaluate publication bias, with a *p*-value < 0.05 considered statistically significant.

## 3 Results

### 3.1 Literature screening and selection

**Figure 1:**
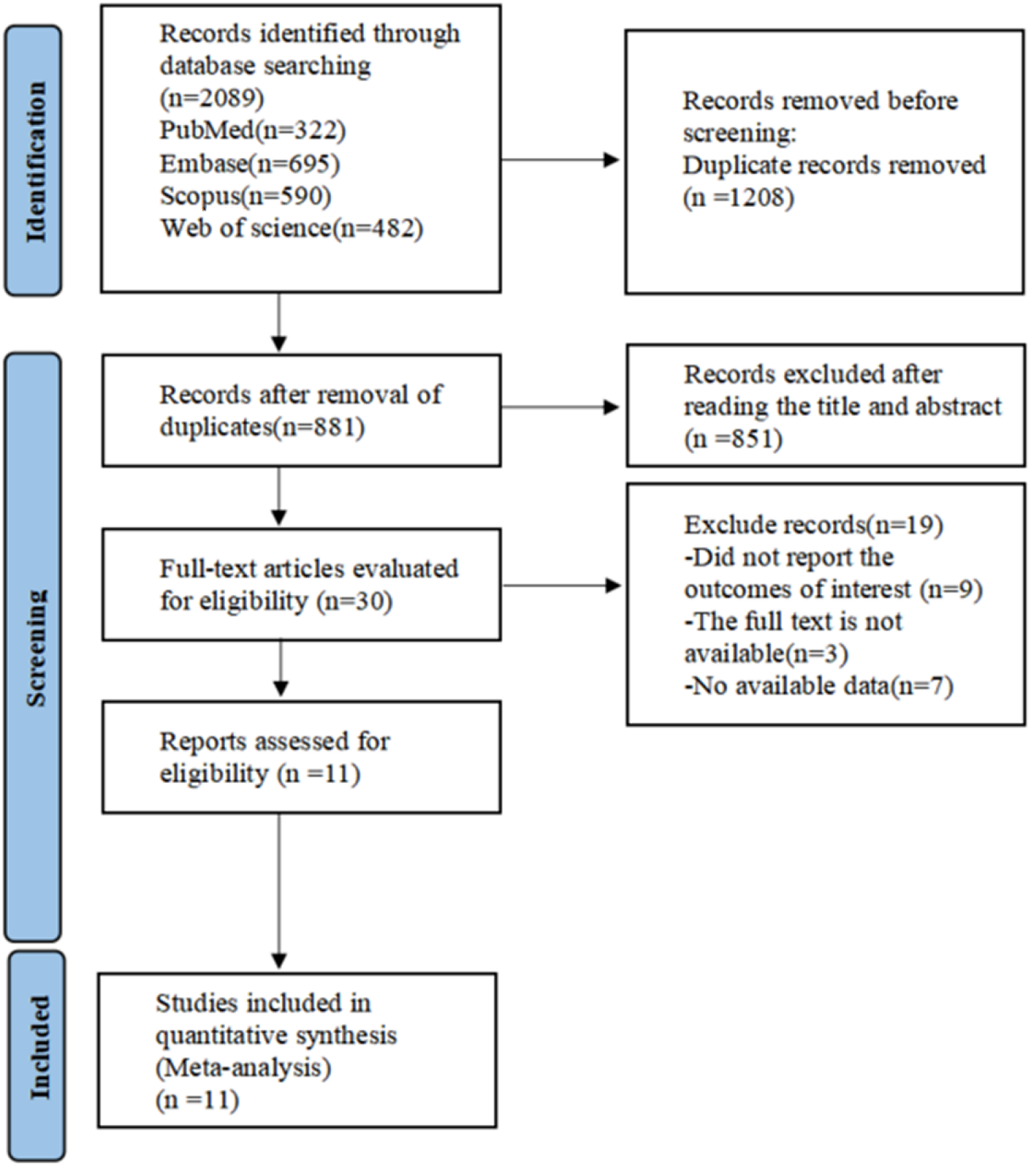
PRISMA flow diagram of the study process. PRISMA, Preferred Reporting Items for Systematic review and Meta-analysis.

The database search initially resulted in 2,089 documents. Following the use of EndNote 20 and manual verification by the researchers, this number was narrowed down to 881 articles. During the preliminary review of titles and abstracts, two researchers identified 30 pertinent articles. Subsequent full-text reviews further reduced the selection down to 11 articles for inclusion. The literature screening process and its outcomes are illustrated in **Figure 1**.

### 3.2 Characteristics and quality of included studies

The meta-analysis included 11 MR studies (29–39). All studies were evaluated as high quality (**Supplementary Table 3**). These studies included subjects from multiple datasets (29–32, 34, 37), covering Europeans (31–36, 38, 39), Africans (29, 37), Asians (29, 30, 37), and Latin Americans (29, 37). Most MR studies used a strict linkage disequilibrium (LD) threshold (R^2^ < 0.001) and restricted the genetic distance to a maximum of 10,000 kilobases (kb) to select independent SNPs as IVs for exposure. However, some studies opted to identify all conditionally independent SNPs in GWAS. The number of SNPs used in the studies varied from tens to thousands, with one study failing to report the number of SNPs used in MR (30). One study did not report the sample size for the outcome variable (39). The most frequently utilized cohort was the UK Biobank, which was featured in seven studies (29, 32, 34, 36, 37, 39). Additionally, four of the cohorts focused on stroke outcomes and all utilized the MEGASTROKE consortium (31, 32, 36–38). Two studies considered stratification by arterial gender (39), and population (30) respectively. All studies were statistically analyzed for MR, with one study reporting results based on IVW, MR-Egger regression, WME, simple mode, and weighted mode (34). Due to the limited number of included studies, funnel plots were not generated for all phenotypes.

### 3.3 Meta-analysis results

A total of four studies on type 2 diabetes mellitus (T2DM) (29, 35–37), two studies on waist-hip ratio (WHR) (29, 32), five studies on BMI (32, 33, 35, 37, 39), five studies on triglycerides (TGs) (six datasets) (29–31, 35, 37), four studies on high-density lipoprotein cholesterol (HDL-C) (five datasets) (30, 31, 35, 37), four studies on low-density lipoprotein cholesterol (LDL-C) (five datasets) (30, 31, 35, 37), four studies on systolic blood pressure (SBP) (29, 32, 35, 36), three studies on diastolic blood pressure (DBP) (29, 35, 36), two studies on hypertension (35–37), two studies on estimated glomerular filtration rate (eGFR) (three datasets) (34, 38), two studies on CKD (three datasets) (34, 38), two studies on smoking (35, 37), and two studies on educational level (35, 36).

#### 3.3.1 Obesity related indicators

**Figure 2:**
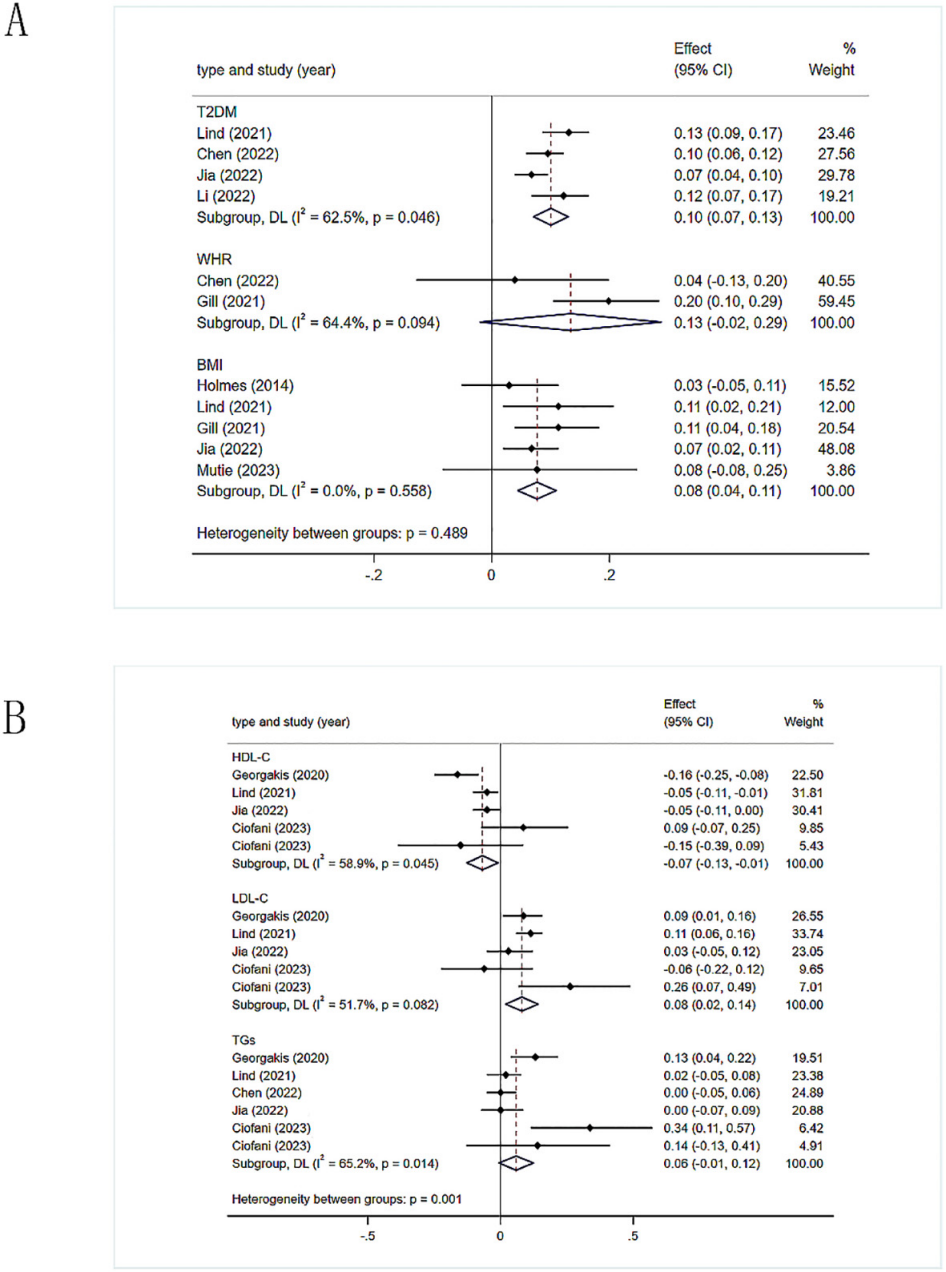
**(A)** Meta-analysis results of the association between genetic liability to obesity-related indicators and risk of stroke. T2DM, type 2 diabetes mellitus; WHR, waist-hip ratio; BMI, body mass index. **(B)** Meta-analysis results of the association between genetic liability to lipid related indicators and risk of stroke. HDL-C, high-density lipoprotein cholesterol; LDL-C, low-density lipoprotein cholesterol; TGs, triglycerides.

The study demonstrated that genetic tendencies towards two obesity-related measures correlated with an increased stroke risk, although this correlation was not observed for one of the measures (**Figure 2**). Specifically, T2DM [1.11(1.07-1.14)], WHR [1.14, (0.98-1.33)], BMI [1.08(1.05-1.12)]. There was heterogeneity between T2DM and stroke (*I^2^ =* 62.5%, *p*=0.046), which we attribute to variations in the demographics of the study populations. Therefore, we utilized a random-effects model. Heterogeneity was also observed in the correlation between WHR and stroke (*I* ^2^ = 64.4%, *p*=0.094). It is possible that differences in the populations used for the exposure factors contributed to this, while the outcome metrics were based on the same population. There was no heterogeneity between BMI and stroke risk when using a fixed-effects model.

#### 3.3.2 Lipid-related Indicators

The IVW method was used to calculate the values, which were then utilized to evaluate the overall causal effect of the three lipid-related indicators on stroke (**Figure 2**). HDL-C has a protective effect against stroke [0.94, (0.97-0.97)], and there was slight heterogeneity in the results (*I* ^2^ = 58.9%, *p*=0.045). The heterogeneity was possibly due to the fact that HDL-C data was derived from five different populations, necessitating the use of a random-effects model. Elevated levels of LDL-C might correlate with an increased likelihood of stroke [1.08(1.05-1.12)], with slight heterogeneity in the results (*I* ^2^ = 51.7%, *p* = 0.082), possibly due to the inclusion of LDL-C data from five different populations, thus requiring a random-effects model. There was no evidence of a causal relationship between TGs and stroke [1.06 (0.99-1.13)].

#### 3.3.3 Blood pressure-related indicators

**Figure 3:**
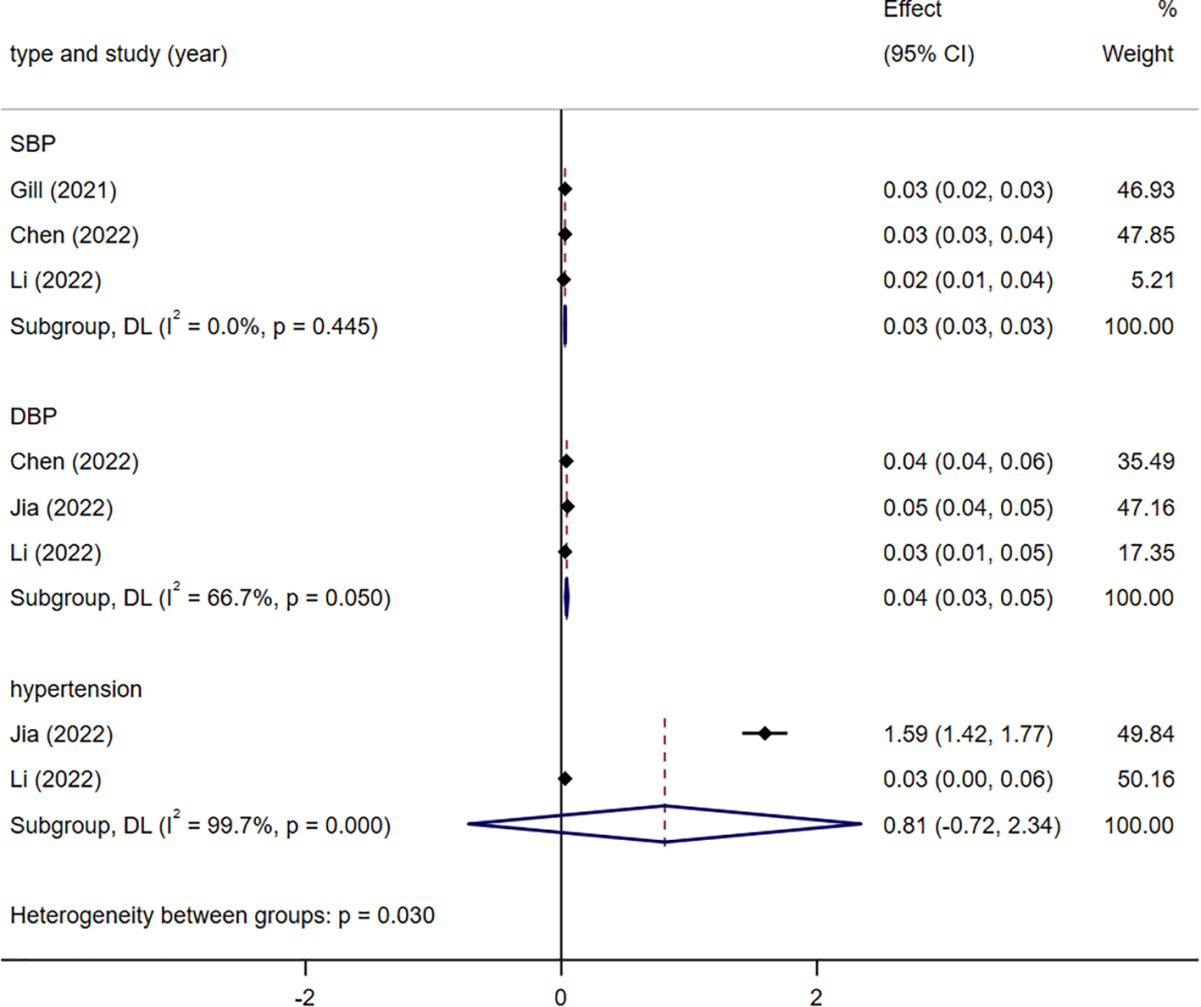
Meta-analysis results of the association between genetic liability to blood pressure-­related indicators and risk of stroke. SBP, systolic blood pressure; DBP, diastolic blood pressure.

Genetic susceptibility to two blood pressure-related indices correlated with a heightened likelihood of developing stroke (**Figure 3**). Specifically, DBP [1.04 (1.03 -1.05)], SBP [1.03 (1.03 -1.03)], and hypertension [2.25(0.49-10.40)]. There was heterogeneity in the statistical results between DBP(*I* ^2^ = 66%), SBP(*I* ^2^ = 0%), hypertension(*I* ^2^ = 99.7%) and stroke. The heterogeneity in the results was attributed to the use of different populations and strata as exposure factors. All analyses were conducted using a random-effects model.

#### 3.3.4 Renal function related indicators

**Figure 4:**
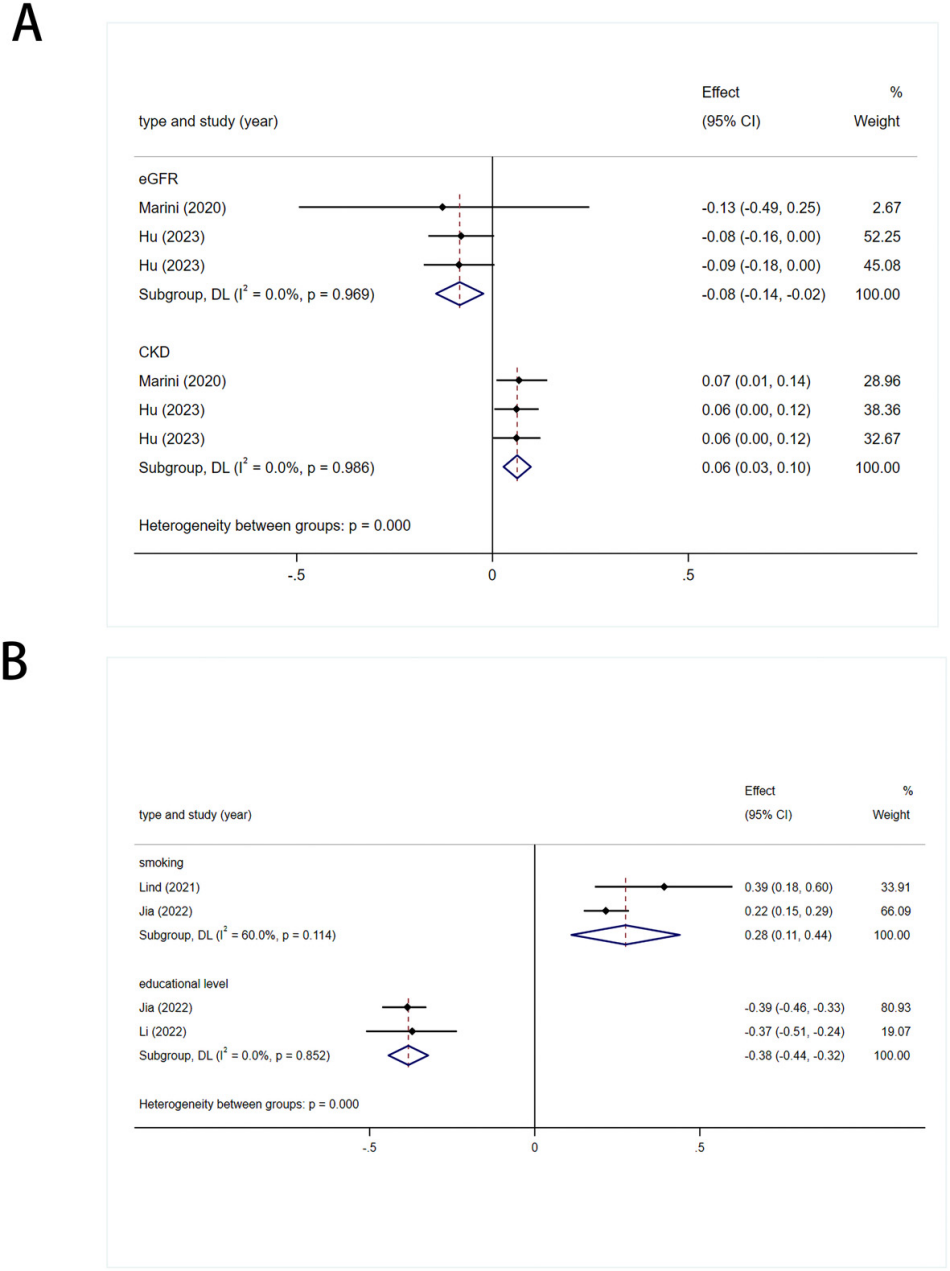
**(A)** Meta analysis results of the association between genetic liability to renal function related indicators and risk of stroke. eGFR, estimated glomerular filtration rate; CKD, chronic kidney disease. **(B)** Meta analysis results of the association between genetic liability to living environment related indicators and risk of stroke.

Values obtained using the IVW method were used to assess the overall causal impact of two renal function-related indicators on stroke: eGFR [0.92 (0.87-0.98)] and CKD [1.07 (1.03-1.10)]. The results suggest a causal effect for both indicators. There was no heterogeneity (*I* ^2^ = 0%; *I* ^2^ = 0%) in the statistical results for the onset of stroke using fixed-effects models (**Figure 4**, **Supplementary Figure 1**).

#### 3.3.5 Living environment-related indicators

A causal link was established between stroke and smoking [1.32 (1.12-1.55)], with slight heterogeneity in the results (*I* ^2^ = 60%, *p*= 0.114), which was attributed to different populations. Therefore, a random-effects model was used. Educational level had a protective effect against stroke [0.68 (0.64-0.72)]. There was no heterogeneity in the results (*I* ^2^ = 0%, *p*= 0.852), so a fixed-effects model was used (**Figure 4**).

#### 3.3.6 Subgroup analoysis of indicators related to ischemic stroke

**Figure 5:**
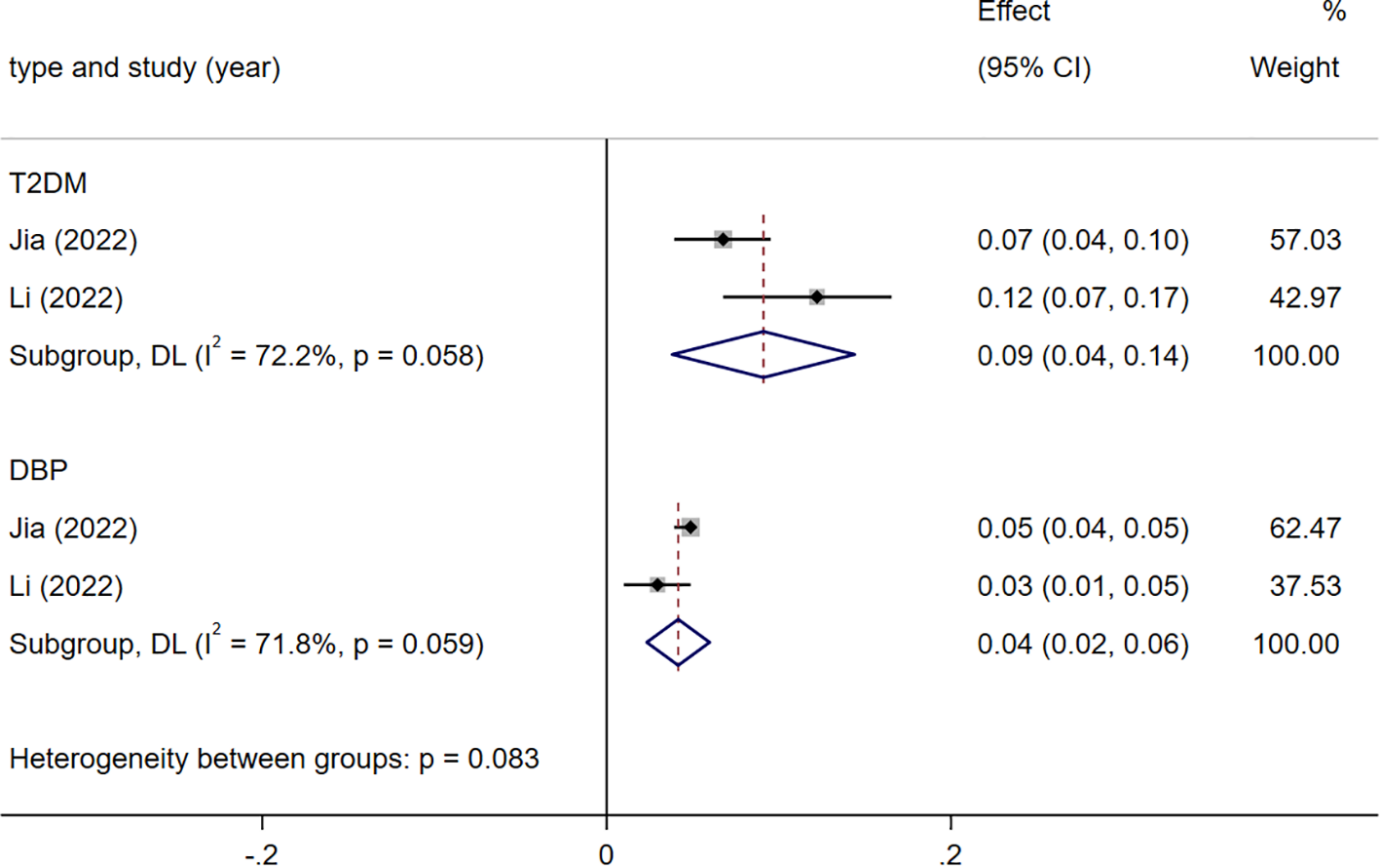
Meta analysis results of indicators related to ischemic stroke(IS). T2DM, type 2 diabetes mellitus; DBP, diastolic blood pressure.

The two indicators associated with IS are T2DM [1.10 (1.04-1.15)], DBP [1.04 (1.02-1.06)]. They all exhibit heterogeneity (*I* ^2^ = 72.2%, *I* ^2^ = 71.8%), attributed to differences in populations and stratification (**Figure 5**).

## 4 Discussion

This review is a meta-analysis of published MR findings related to stroke. Genetic evidence suggests that T2DM, BMI, LDL-C, SBP, DBP, CKD, and smoking contribute to a higher likelihood of stroke, while HDL-C, eGFR, and educational level are protective factors against stroke. Conversely, no causal link was found between WHR, TGs, and stroke. In subgroup analyses, T2DM and DBP showed a correlation with a higher risk of IS.

### 4.1 Obesity related indicators

Obesity markedly elevates the likelihood of developing cardiovascular disease by inducing metabolic syndrome, which involves insulin resistance, elevated blood glucose levels, substantial body fat accumulation, irregular cholesterol profiles, and hypertension (40, 41). Specifically, in visceral obesity, the combined effects of adipocyte hypertrophy and proliferation lead to an elevated release of pro-inflammatory cytokines (42), exacerbating the systemic and vascular inflammatory response (43–45). Further supporting this, our meta-analysis revealed that obesity-related indicators, such as BMI and T2DM, are risk factors for stroke, consistent with experimental findings. Notably, a higher BMI was correlated with a heightened overall risk of stroke, with combined RR values of 1.25 (95% CI 1.16-1.34, *I* ^2^ = 84.8%, *p* = 0.00) and 1.47 (95% CI 1.02-2.11, *I* ^2^ = 99.4%, *p* = 0.04) (46–48). Moreover, T2DM was found to be causal for stroke in Europeans (49) (95% CI 1.06-1.09). While studies have shown that WHR is a risk factor for stroke (50–52), intriguingly, our meta-analysis indicated no direct causal relationship between WHR and stroke. This might be due to gender differences in how visceral fat relates to calcified atherosclerosis (53–57). The differing findings between our study and Riaz H et al. may be due to differences in study populations and genetic instruments used (58). Our analysis includes a broader population and more recent studies, which may provide updated data. Methodological variations, such as inclusion criteria and statistical approaches, could also contribute to the discrepancies. Therefore, we propose that for a more comprehensive anthropometric assessment of cardiovascular disease(CVD) risk, a combination of measurements including WHR, WC, and Waist-to-Height Ratio (WHtR) should be utilized (59–61).

### 4.2 Lipid-related indicators

According to our systematic meta-analysis and comprehensive observational studies, elevated levels of HDL-C are significantly associated with the prevention of IS. Concurrently, LDL-C has been identified as the most useful biomarker for predicting the risk of stroke (62–65). HDL-C facilitates the uptake of cholesterol from peripheral tissues through the reverse cholesterol transport (RCT) pathway. It influences macrophage activity and function, triggers local aggregation of pro-inflammatory cells (66–68), and its antioxidant properties prevent the oxidation of LDL into ox-LDL, thus inhibiting the process of atherosclerosis formation (69). However, our meta-analysis indicates no definitive evidence supporting a causal relationship between TGs and stroke. Research indicates arteriosclerosis-inducing dyslipidemia is attributed to other lipid components (70, 71), notably triglyceride-rich lipoproteins (TRLs) and remnant cholesterol (72), which are crucial in atherosclerotic processes (70, 73). Therefore, further investigation into the collective influence of TGs, TRLs, and remnant cholesterol on atherosclerotic cardiovascular disease (ASCVD) is warranted.

### 4.3 Blood pressure-related indicators

Studies have confirmed that SBP, DBP, and hypertension are risk factors for stroke, a finding supported by our meta-analysis (74–77). In endothelial cells, a deficiency in Piezo1 can impair flow-mediated vasodilation and elevate SBP (78). Additionally, endothelial damage (79), proliferation of vascular smooth muscle cells (VSMCs) (80, 81), and infiltration of immune cells (82), may induce hypertension (83). Reduced expression of transmembrane member 16A (TMEM16A) may promote cellular proliferation and brain vascular remodeling induced by hypertension (84). Furthermore, the China Stroke Primary Prevention Trial (CSPPT) demonstrated that the risk of stroke was lowest in patients with an average SBP of 120‐130 mm Hg, increasing in those with SBP <120 mm Hg and SBP 130-140 mmHg (85). Therefore, we suggest enhancing awareness and improving treatment compliance for hypertension to effectively prevent stroke.

### 4.4 Renal function related indicators

Our meta-analysis and systematic review indicate CKD as a significant risk factor for stroke (86–89). This is further supported by studies demonstrating CKD’s independent impact on the risk of stroke, especially when characterized by reduced eGFR (34, 89). Moreover, eGFR is crucial for assessing renal function, with levels < 15, 15-29, and 30-44 mL/min/1.73m^2^ closely linked to increased likelihoods of adverse clinical outcomes in stroke patients (90–93). However, research on the categorization of eGFR in relation to stroke prognosis (90). Therefore, our study emphasizes the importance of monitoring renal function in these patients (93). We advocate for further research to investigate the temporal relationship between eGFR and stroke over time, and we recommend eGFR as the preferred marker for assessing stroke risk.

### 4.5 Living environment-related indicators

Previous observational studies, along with our meta-analysis, indicate that smoking and lower educational levels increase the risk of stroke (74, 94, 95). Delgado et al. discovered elevated levels of soluble Intercellular Adhesion Molecule-1 (sICAM-1), soluble Vascular Cell Adhesion Molecule-1 (sVCAM-1), as well as sE-selectin, sP-selectin, and sL-selectin in smokers. These elements are produced by both endothelial cells and leukocytes (96). Consequently, we believe that smoking cessation should be a primary intervention. Additionally, since higher educational attainment is linked to a decreased risk of stroke (97, 98), we recommend improving the overall education and cultural literacy of the general population.

### 4.6 Indicators related to ischemic stroke

Subgroup analysis of IS indicated that T2DM, DBP significantly elevate IS risk, consistent with clinical observations. However, our analysis did not find evidence of a causal relationship between SBP and IS, this result we view with caution. Considering clinical studies affirming the association of IS with SBP (74), we still advise IS patients to diligently monitor both SBP and DBP, engage in moderate exercise, and actively manage blood glucose and BP. Although limited clinical studies exist in this area, some research does support a causal relationship between BP and SV-Stroke (36, 99). Therefore, we recommend observational studies analyzing risk factors for stroke subtypes (large artery, cardioembolic, SV-Stroke) to further understand these associations.

## 5 Clinical implications and future research

Given the heightened risk of stroke associated with obesity, abnormal BP, and smoking, it is crucial to prioritize the promotion of healthy dietary habits, regular physical activity, and stress reduction. Additionally, the implementation of smoking cessation programs is essential for primary prevention and non-pharmacological interventions. Enhancing educational and literacy levels is also key in reducing the occurrence of strokes. While our findings show no clear association between WHR and stroke, we remain skeptical of these results. Therefore, it is still recommended to maintain a healthy body weight and closely monitor metrics such as WHR, WC, and WHtR.

This paper summarizes current MR research on risk factors for stroke but identifies several issues that need further study. Specifically, it emphasizes the importance of incorporating novel SNPs as IVs in stroke etiology research through MR studies. This method leverages recent genetic markers to more precisely determine the influence of diverse risk factors on stroke. Further research should expand to encompass diverse cohorts, especially those exposed to different environmental factors, for a more comprehensive understanding of stroke risk factors. The MR method is crucial in providing new insights into epidemiological studies and elucidating complex diseases such as stroke, including their pathophysiology and pharmacological treatments. Future efforts should focus on integrating MR into clinical settings to improve treatment protocols and reduce medication side effects. This advancement could significantly enhance stroke prevention and therapy.

This meta-analysis has several limitations. Firstly, the limited number of existing MR studies on stroke restricts our ability to assess publication bias through funnel plot symmetry analysis and the application of Egger’s and Begg’s tests. Additionally, we were unable to perform subgroup analyses based on region, age, and sex, limiting our exploration of the potential effects of these variables on the consolidated results. Secondly, the significant heterogeneity observed across studies necessitates careful interpretation of the results. This heterogeneity is somewhat expected given the variations in study methods, participant characteristics, and locations. In summary, although MR methods offer advantages over traditional meta-analysis and provide strong evidence linking stroke with its risk factors, they still have limitations. MR studies may be affected by measurement errors in exposure and outcomes, as well as limited ability to capture longitudinal causal relationships.

## 6 Conclusion

In conclusion, risk factors for stroke include obesity, dyslipidemia, abnormal BP, CKD, and smoking. Conversely, HDL-C, eGFR, and higher levels of education serve as protective factors against stroke. Therefore, the likelihood of stroke can be significantly reduced by quitting smoking, maintaining a healthy body weight, addressing CKD treatment, and improving educational attainment, particularly in individuals predisposed to stroke, such as hereditary susceptibility.

## Data Availability

The original contributions presented in the study are included in the article/**Supplementary Material**. Further inquiries can be directed to the corresponding author.

## Supplementary material

Supplementary Figure 1
**(A)** Meta-analysis results of the association between genetic liability to chronic kidney disease (CKD) and risk of stroke. CKD: chronic kidney disease; IVW: inverse variance weighted, MR-Egger: MR-Egger regression; simple mode; weighted mode; WME: weighted median estimator. **(B)** Meta-analysis results of the association between genetic liability to estimated glomerular filtration rate (eGFR) and risk of stroke. CKD: chronic kidney disease; IVW: inverse variance weighted, MR-Egger: MR-Egger regression; simple mode; weighted mode; WME: weighted median estimator.
