# Supplementary figures and images for: Mendelian randomization studies of lifestyle-related risk factors for stroke: a systematic review and meta-analysis
Source: Front Endocrinol (Lausanne). 2024 Nov 4;15:1379516. doi: 10.3389/fendo.2024.1379516 (PMC11570884; doi:10.3389/fendo.2024.1379516)

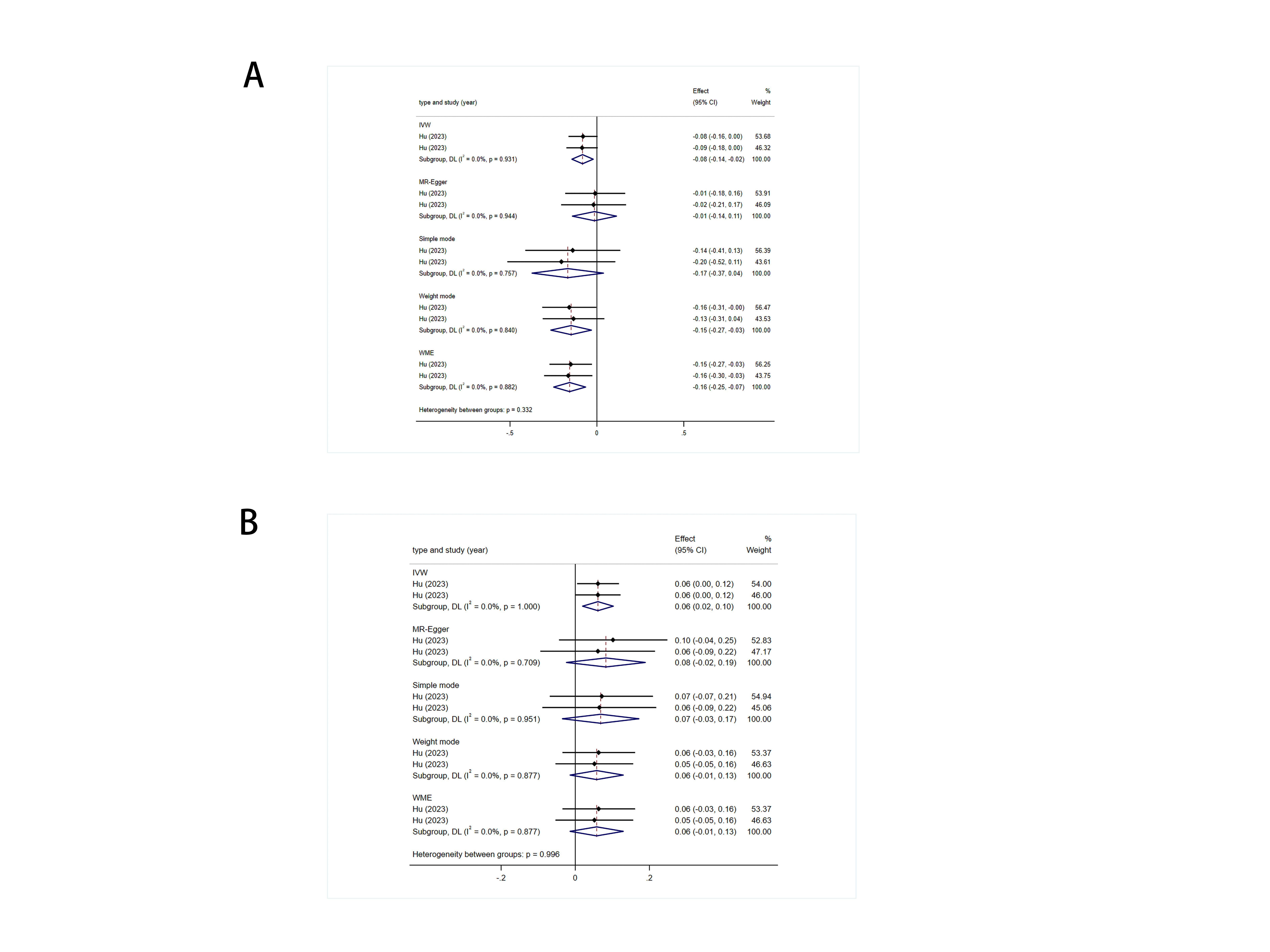

Supplement: Supplementary Figure 1 — (A) Meta-analysis results of the association between genetic liability to chronic kidney disease (CKD) and risk of stroke. CKD: chronic kidney disease; IVW: inverse variance weighted, MR-Egger: MR-Egger regression; simple mode; weighted mode; WME: weighted median estimator. (B) Meta-analysis results of the association between genetic liability to estimated glomerular filtration rate (eGFR) and risk of stroke. CKD: chronic kidney disease; IVW: inverse variance weighted, MR-Egger: MR-Egger regression; simple mode; weighted mode; WME: weighted median estimator. [file Image1.tif]
