# Supplementary material for: Mendelian randomization studies of lifestyle-related risk factors for stroke: a systematic review and meta-analysis
Source: Front Endocrinol (Lausanne). 2024 Nov 4;15:1379516. doi: 10.3389/fendo.2024.1379516 (PMC11570884; doi:10.3389/fendo.2024.1379516)
Supplement: Supplementary file 2 [file Table1.doc]

Supplementary Table i. searching from four databases: PubMed, Web of Science, Embase, and Scopus

1.Pubmed

search formula：

((((((((("Stroke"[Mesh]) OR (Stroke[Title/Abstract])) OR (Brain Infarction[Title/Abstract])) OR (Brain Stem Infarctions[Title/Abstract])) OR (Cerebral Infarction[Title/Abstract])) OR (Hemorrhagic Stroke[Title/Abstract])) OR (Ischemic Stroke[Title/Abstract])) OR (Embolic Stroke[Title/Abstract])) OR (Thrombotic Stroke[Title/Abstract])) AND (("Mendelian Randomization Analysis"[Mesh]) OR (Mendelian Randomization Analysis[Title/Abstract]))

2.Web of science

search formula：

((((((((((((TS=(Stroke)) OR TS=(Stroke)) OR TS=(Brain Infarction)) OR TS=(Brain Stem Infarctions )) OR TS=(Cerebral Infarction )) OR TS=(Hemorrhagic Stroke)) OR TS=(Ischemic Stroke)) OR TS=(Embolic Stroke))) OR TS=(Thrombotic Stroke))) AND TS=(Mendelian Randomization Analysis))

3.Embase

session results：

No. Query Results Results Date

#15. #11 AND #14 695 7 Aug 2023

#14. #12 OR #13 8,200 7 Aug 2023

#13. 'mendelian randomization analysis':ab,ti 1,178 7 Aug 2023

#12. 'mendelian randomization analysis'/exp 8,067 7 Aug 2023

#11. #1 OR #2 OR #3 OR #4 OR #5 OR #6 OR #7 OR #8 OR 626,018 7 Aug 2023

#9 OR #10

#10. 'thrombotic stroke':ab,ti 549 7 Aug 2023

#9. 'embolic stroke':ab,ti 4,257 7 Aug 2023

#8. 'ischemic stroke':ab,ti 105,534 7 Aug 2023

#7. 'hemorrhagic stroken':ab,ti 7 Aug 2023

#6. 'cerebral infarction':ab,ti 24,728 7 Aug 2023

#5. 'brain stem infarctions':ab,ti 40 7 Aug 2023

#4. 'brain infarction':ab,ti 3,585 7 Aug 2023

#3. 'stroke':ab,ti 485,185 7 Aug 2023

#2. 'cerebrovascular accident':ab,ti 8,582 7 Aug 2023

#1. 'cerebrovascular accident'/exp 428,058 7 Aug 2023

4.scoupus

search formula:

(TITLE-ABS-KEY(Stroke) OR TITLE-ABS-KEY(Brain Infarction) OR TITLE-ABS-KEY(Brain Stem Infarctions) OR TITLE-ABS-KEY(Cerebral Infarction) OR TITLE-ABS-KEY(Hemorrhagic Stroke) OR TITLE-ABS-KEY(Ischemic Stroke) OR TITLE-ABS-KEY(Embolic Stroke) OR TITLE-ABS-KEY(Thrombotic Stroke) AND TITLE-ABS-KEY(Mendelian Randomization Analysis))
