# Supplementary material for: Mendelian randomization studies of lifestyle-related risk factors for stroke: a systematic review and meta-analysis
Source: Front Endocrinol (Lausanne). 2024 Nov 4;15:1379516. doi: 10.3389/fendo.2024.1379516 (PMC11570884; doi:10.3389/fendo.2024.1379516)
Supplement: Supplementary file 3 [file Table2.doc]

| Supplementary Table ii.Study characteristics of all 11 studies included for qualitative analysis | Name | Hu^34^ | Chen^29^ | Jia^35^ | Li^36^ | Lind^37^ | Gill^32^ | Georgakis^31^ | Holmes^33^ | Mutie^39^ | Ciofani^30^ | Marini^38^ |
| --- | --- | --- | --- | --- | --- | --- | --- | --- | --- | --- | --- | --- |
|  | Year | 2023 | 2022 | 2022 | 2022 | 2021 | 2021 | 2020 | 2014 | 2023 | 2023 | 2020 |
|  | Ethnicity | European | European; African ancestry; Asian ancestry; Latin American | European | European | African;European;Asian;Latin American | European;86% European-ancestry | European | European | white European | East Asian;European | European |
|  | Cohort | CKDGen; Pan-UK Biobank; MVP; PAGE; SUMMIT | DIAGRAM;GERA;UKB Biobank;ICBP | IEU OpenGWAS ; UK Biobank | UK Biobank;MEGASTROKE | GLGC; GIANT consortium; DIAGRAM; UK biobank; METASTROKE | GIANT consortium; UK Biobank; MEGASTROKE; DIAGRAM; GLGC | MVP; GLGC; MEGASTROKE | ARIC; CHS; CARDIA; EPIC-NL; FHS; MEDAL; MESA;WHI | UK Biobank | GLGC | CKDGen; MEGASTROKE |
|  | Design of MR Study | two-sample | two-sample | two-sample | two-sample | two-sample | two-sample | two-sample | two-sample | two-sample | two-sample | two-sample |
|  | Exposure | eGFR;CKD | T2DM;TG;WHR;SBP;DBP; | 2DM;TG;HDL-C;BMI;LDL-C;Smoking;SBP;DBP;hypertension;Educational level | SBP;DBP;hypertension;Educational level;2DM; | 2DM;TG;HDL-C;BMI;LDL-C;Smoking | BMI;WHR;SBP | TG;HDL-C;LDL-C | 2DM;BMI | BMI（Men;Women） | TG;HDL-C;LDL-C | eGFR;CKD |
|  | Sample Size | 40,585 cases, 406,111 controls(stroke), 34,217 cases , 406,111 controls(ischemic stroke) | 67,162 cases, 454,450 controls | 40,585 cases, 406,111 controls | 40,585 cases, 406,111 controls | 67,162 cases, 454,450 controls | 67,162 cases, 454,450 controls | 11,710 cases, 287,067 controls | 3,813cases, 23,782 controls | / | 34,217 cases, 406,111 controls | 40,585 cases, 406,111 controls |
|  | Principal findings | The evidence to support the causal effects of kidney function on CVDs is currently insufficient | SBP; DBP; and TG mediate the causal effect of T2DM on CVD | 11 modifiable factors are important targets for preventing CVD | The study identified exposome elements causally related to IS and its subtypes | SBP was causally related to all four cardiovascular outcomes | reducing obesity will lower the risk of cardiovascular disease metabolic risk factors | Raising HDL-C could be considered for the prevention of SV-Stroke | Identified causal effects of BMI on several cardiometabolic traits | the shapes of causal effects of BMI on cardiometabolic diseases | This study provides evidence for causal relations between cardiovascular risk factors and IHD and AF | Impaired kidney function may be causally involved in the pathogenesis of LAS |
