# Supplementary material for: Mendelian randomization studies of lifestyle-related risk factors for stroke: a systematic review and meta-analysis
Source: Front Endocrinol (Lausanne). 2024 Nov 4;15:1379516. doi: 10.3389/fendo.2024.1379516 (PMC11570884; doi:10.3389/fendo.2024.1379516)
Supplement: Supplementary file 4 [file Table3.doc]

| Supplementary Table iii. Quality Assessment tool conducted based on adherence to the Strengthening the Reporting of Mendelian Randomization Studies (STROBE-MR) Guidelines for all 11 studies included in qualitative analysis. Each item is scored between 0 and 1 for each criterion to yield a total score. Upon conversion of the quality assessment score to a percentage, scores of < 75%, 75–85% and > 85% were considered to indicate high, medium and low risk of bias, respectively. | Study and Year of Publication | ^34^Hu et al, 2023 | ^29^Chen et al, 2022 | ^35^Jia et al, 2022 | ^36^Li et al, 2022 | ^37^Lind et al, 2021 | ^32^Gill et al, 2021 | ^31^Georgakis et al, 2020 | ^33^Holmes et al, 2014 | ^39^Mutie et al, 2023 | ^30^Ciofani et al, 2023 | ^38^Marini et al,2020 |
| --- | --- | --- | --- | --- | --- | --- | --- | --- | --- | --- | --- | --- |
|  | Title and Abstract | 1 | 1 | 1 | 1 | 1 | 1 | 1 | 1 | 1 | 1 | 1 |
|  | Background | 1 | 1 | 1 | 1 | 1 | 1 | 1 | 1 | 1 | 1 | 1 |
|  | Objectives | 1 | 1 | 1 | 1 | 1 | 1 | 1 | 1 | 1 | 1 | 1 |
|  | Study Design and Data Sources | 1 | 1 | 1 | 1 | 0.5 | 1 | 1 | 1 | 0.5 | 1 | 1 |
|  | Statistical Methods:Main Analysis | 1 | 1 | 1 | 1 | 1 | 1 | 1 | 1 | 1 | 1 | 1 |
|  | Software and Pre-Registration | 1 | 1 | 1 | 1 | 1 | 1 | 1 | 1 | 1 | 1 | 1 |
|  | Descriptive Data | 1 | 1 | 1 | 1 | 1 | 1 | 1 | 1 | 1 | 1 | 1 |
|  | Main Results | 1 | 1 | 1 | 1 | 1 | 1 | 1 | 1 | 1 | 1 | 1 |
|  | Sensitivity and Additional Analysis | 1 | 1 | 1 | 0.5 | 1 | 1 | 1 | 1 | 1 | 0.5 | 1 |
|  | Key Results | 1 | 1 | 1 | 1 | 1 | 1 | 1 | 1 | 1 | 1 | 1 |
|  | Limitations | 1 | 1 | 1 | 1 | 1 | 1 | 0.5 | 1 | 0.5 | 0.5 | 1 |
|  | Interpretation | 1 | 1 | 1 | 1 | 1 | 1 | 1 | 1 | 1 | 1 | 1 |
|  | Generalizability | 1 | 1 | 1 | 1 | 1 | 1 | 1 | 1 | 1 | 1 | 1 |
|  | MR Core Assumptions | 1 | 1 | 1 | 1 | 1 | 1 | 1 | 1 | 1 | 1 | 1 |
|  | Total Score(out of 14) | 14 | 14 | 14 | 13.5 | 13.5 | 14 | 13.5 | 14 | 13 | 13 | 14 |
|  | Score(%) | 100.0 | 100.0 | 100.0 | 96.4 | 96.4 | 100.0 | 96.4 | 100.0 | 92.9 | 92.9 | 100.0 |
